# Supplementary material for: Plasmodium ARK1 regulates spindle formation during atypical mitosis and forms a divergent chromosomal passenger complex
Source: Nat Commun. 2026 Feb 26;17:1598. doi: 10.1038/s41467-026-69460-7 (PMC12946202; doi:10.1038/s41467-026-69460-7)
Supplement: Supplementary file 2 — Description Of Additional Supplementary File [file 41467_2026_69460_MOESM2_ESM.pdf]

Description of additional supplementary files

Supplementary Data 1. List of genes identified as differentially expressed in *P. berghei ark1PTD* gametocytes compared to wild-type (WT-GFP) controls.

Two tabs in the spreadsheet: Tab 1: non-activated gametocytes, Tab 2: 30 min post-activation gametocytes. Differential gene expression analysis was performed using the DESeq2 package. Statistical significance was determined using a two-sided Wald test, with *P*-values adjusted for multiple comparisons using the Benjamini-Hochberg procedure.

Supplementary Data 2. Protein identifications for LC-MS/MS for *P. berghei* ARK1-GFP and WT-GFP.

Four tabs in the spreadsheet: Tab 1: data information, Tab 2: volcano plot data, Tab 3: schizonts (8-9 h) WT vs. ARK1-GFP data, Tab 4: male gametocytes (1.5 min) WT vs. ARK1-GFP data.

Supplementary Data 3. PCR primer used in the *P. falciparum* and *P. berghei* study.

Two tabs in the spreadsheet: Tab 1: primers used in the *P. falciparum* study, Tab 2: primers used in the *P. berghei* study.

Supplementary Data 4. Primary, secondary antibodies and stains used in the *P. falciparum* and P. *berghei* study.

Two tabs in the spreadsheet: Tab 1: primary, secondary antibodies and stains used in the *P. falciparum* study, Tab 2: primary, secondary antibodies and stains used in the *P. berghei* study.

Supplementary Data 5. Proteome sources and amino acid sequences of Aurora and CPC used during this study.

Three tabs in the spreadsheet: Tab 1: data information, Tab 2: sources for predicted proteome database, Tab 3: number and amino acid sequences for ARK1/Aurora, ARK2, ARK3, INCENP-A/-B, borealin, survivin genes across eukaryotes with a focus on alveolata, apicomplexa.

Supplementary Movie 1. Time-lapse video microscopy of gametogony in the Hoechs t (blue)-stained PbARK1-GFP (green) male gametocyte after 2-3 min activation.

Video is played back at 15× speed. Scale bar = 2 μm.

Supplementary Movie 2. Time-lapse video microscopy of gametogony in the Hoechst (blue)-stained PbARK1-GFP (green) male gametocyte after 4-5 min activation.

Video is played back at 15× speed. Scale bar = 2 μm.

Supplementary Movie 3. Time-lapse video microscopy of gametogony in the PbARK1-GFP (green) male gametocyte after 6-8 min activation.

Video is played back at 15× speed. Scale bar = 2 μm.

Supplementary Movie 4. Time-lapse video microscopy of gametogony in the PbARK1-GFP (green) and PbNDC80-mCherry (magenta) male gametocyte after 4-5 min activation.

Video is played back at 15× speed. Scale bar = 2 μm.

Supplementary Movie 5. Time-lapse video microscopy of gametogony in the PbARK1-GFP (green) and PbARK2-mCherry (magenta) male gametocyte after 2-3 min activation.

Video is played back at 15× speed. Scale bar = 2 μm.

Supplementary Movie 6. Time-lapse video microscopy to assess egress of DMSO-treated HA-PfARK1-loxP parasites.

Related to Fig. 4g(i).

Supplementary Movie 7. Time-lapse video microscopy assess egress of RAP-treated HA-PfARK1-loxP parasites.

Related to Fig. 4g(ii).

Supplementary Movie 8. Time-lapse video microscopy assess egress of RAP-treated HA-PfARK1-loxP parasites.

Related to Fig. 4g(iii).
