# Supplementary material for: Plasmodium ARK1 regulates spindle formation during atypical mitosis and forms a divergent chromosomal passenger complex
Source: Nat Commun. 2026 Feb 26;17:1598. doi: 10.1038/s41467-026-69460-7 (PMC12946202; doi:10.1038/s41467-026-69460-7)
Supplement: Supplementary file 16 — Reporting summary [file 41467_2026_69460_MOESM16_ESM.pdf]

nature portfolio

Corresponding author(s): Rita Tewari, Pushkar Sharma, Eelco C. Tromer

Last updated by author(s): Jan 23, 2026

Reporting Summary

Nature Portfolio wishes to improve the reproducibility of the work that we publish. This form provides structure for consistency and transparency in reporting. For further information on Nature Portfolio policies, see our Editorial Policies and the Editorial Policy Checklist.

Statistics

For all statistical analyses, confirm that the following items are present in the figure legend, table legend, main text, or Methods section.

n/a	Confirmed	
☐	☒	The exact sample size (*n*) for each experimental group/condition, given as a discrete number and unit of measurement
☐	☒	A statement on whether measurements were taken from distinct samples or whether the same sample was measured repeatedly
☐	☒	The statistical test(s) used AND whether they are one- or two-sided<br>*Only common tests should be described solely by name; describe more complex techniques in the Methods section.*
☒	☐	A description of all covariates tested
☒	☐	A description of any assumptions or corrections, such as tests of normality and adjustment for multiple comparisons
☐	☒	A full description of the statistical parameters including central tendency (e.g. means) or other basic estimates (e.g. regression coefficient) AND variation (e.g. standard deviation) or associated estimates of uncertainty (e.g. confidence intervals)
☐	☒	For null hypothesis testing, the test statistic (e.g. *F*, *t*, *r*) with confidence intervals, effect sizes, degrees of freedom and *P* value noted<br>*Give P values as exact values whenever suitable.*
☒	☐	For Bayesian analysis, information on the choice of priors and Markov chain Monte Carlo settings
☒	☐	For hierarchical and complex designs, identification of the appropriate level for tests and full reporting of outcomes
☒	☐	Estimates of effect sizes (e.g. Cohen's *d*, Pearson's *r*), indicating how they were calculated

Our web collection on statistics for biologists contains articles on many of the points above.

Software and code

Policy information about availability of computer code

Data collection	-All the wide field microscopy images were captured using a 63x oil immersion objective or 10x objective on a Zeiss Axio Imager M2 or Z1 microscope fitted with an AxioCam ICc1 digital camera.<br>-UExM images were acquired on a Zeiss Celldiscoverer 7 or a Zeiss LSM980 confocal microscopy.<br>-SIM images were captured with an inverted microscope using Zeiss Plan-Apochromat 63x/1.4 Oil immersion or Zeiss C-Apochromat 63×/1.2 W Korr M27 water immersion objective on a Zeiss Elyra PS.1 microscope, using the structured illumination microscopy (SIM) technique.<br>-The tryptic peptides were analysed by liquid chromatography–tandem mass spectrometry.<br>-Library for RNA seq was sequenced using an Illumina Hiseq 4000 platform (Illumina).
Data analysis	-All the wide field microscopy images and U-ExM images were analysed with Fiji (version 1.54f), Zeiss Axio Vision 4.8.3, or Zeiss ZEN 3.1 or 3.9.<br>-Processing and export of SIM images and videos were done by Zeiss Zen 2012 Black edition, Service Pack 5 and Zeiss Zen 2.1 Blue edition.<br>-Mascot (http://www.matrixscience.com/) and MaxQuant (https://www.maxquant.org/) search engines were used for mass spectrometry data analysis.<br>-Flow cytometry data was analyzed by using FLowJoV.10.<br>-FastQC (https://www.bioinformatics.babraham.ac.uk/projects/fastqc/), was used to analyze the raw read quality of RNA seq library. trimming of low-quality reads and adapter sequences was done using Trimmomatic. Reads were mapped to the P. berghei ANKA genome (PlasmoDB release 40) using HISAT2 v2.1.0, and gene counts were generated with FeatureCounts. Data were normalized using the TMM method (EdgeR), transformed with voom (limma), and analysed for differential expression using DESeq2. Gene ontology enrichment was done using R package topGO (https://bioconductor.org/packages/release/bioc/html/topGO.html).<br>- All statistical analyses were performed using GraphPad Prism 8.02 or 9 (GraphPad Software) or Excel (Microsoft).

For manuscripts utilizing custom algorithms or software that are central to the research but not yet described in published literature, software must be made available to editors and reviewers. We strongly encourage code deposition in a community repository (e.g. GitHub). See the Nature Portfolio guidelines for submitting code & software for further information.

Data

Policy information about availability of data

All manuscripts must include a data availability statement. This statement should provide the following information, where applicable:

- Accession codes, unique identifiers, or web links for publicly available datasets
- A description of any restrictions on data availability
- For clinical datasets or third party data, please ensure that the statement adheres to our policy

The PlasmoDB database was used for protein annotation identified by mass spectrometry for proteomic studies and the data is submitted to PRIDE (PXD068821), and RNA seq data are submitted to NCBI (PRJNA1309997) , and all raw data are available.

Research involving human participants, their data, or biological material

Policy information about studies with human participants or human data. See also policy information about sex, gender (identity/presentation), and sexual orientation and race, ethnicity and racism.

Reporting on sex and gender	NA
Reporting on race, ethnicity, or other socially relevant groupings	NA
Population characteristics	NA
Recruitment	NA
Ethics oversight	NA

Note that full information on the approval of the study protocol must also be provided in the manuscript.

Field-specific reporting

Please select the one below that is the best fit for your research. If you are not sure, read the appropriate sections before making your selection.

☒ Life sciences ☐ Behavioural & social sciences ☐ Ecological, evolutionary & environmental sciences

For a reference copy of the document with all sections, see nature.com/documents/nr-reporting-summary-flat.pdf

Life sciences study design

All studies must disclose on these points even when the disclosure is negative.

Sample size	The sample size (n) for each experiment is noted within the respective figure legends along with information related to statistical analysis. Typically, 3 or more biological replicates were used.
Data exclusions	None
Replication	Most experiments were done at least three times with reproducible results from each experiment. Wherever possible data was also quantified and appropriate statistical analysis was used to interpret the results.
Randomization	All data collection was randomised and separated by experimental treatments.
Blinding	No blinding occured during these studies.

Reporting for specific materials, systems and methods

We require information from authors about some types of materials, experimental systems and methods used in many studies. Here, indicate whether each material, system or method listed is relevant to your study. If you are not sure if a list item applies to your research, read the appropriate section before selecting a response.

Materials & experimental systems

n/a	Involved in the study
☐	☒ Antibodies
☐	☒ Eukaryotic cell lines
☒	☐ Palaeontology and archaeology
☐	☒ Animals and other organisms
☒	☐ Clinical data
☒	☐ Dual use research of concern
☒	☐ Plants

Methods

n/a	Involved in the study
☒	☐ ChIP-seq
☐	☒ Flow cytometry
☒	☐ MRI-based neuroimaging

Antibodies

Antibodies used	Most antibodies used were from commercial sources or were generated in Sharma's lab. The information is provided in Supplementary Table S4.<br>Anti-HA, 12CA5, mouse antibody (Sigma-11583816001)<br>Anti-HA, C29F4, mouse antibody (Cell Signaling Technology, 3724)<br>Anti-PfGAP45, rabbit antibody (Generated in Dr Shama laboratory)<br>Anti-centrin, clone 20H5, mouse antibody(Sigma-Aldrich, 630249)<br>Anti-α-tubulin, B-5-1-2, mouse antibody (Invitrogen, 32-2500)<br>Anti-α-tubulin, rabbit antibody (Sigma, SAB3501072)<br>Anti-MSP1, rabbit antibody (BEI Resources)<br>β-Actin, mouse HRP conjugated antibody (Santacruz)<br>Anti-BIP, rabbit antibody (BEI Resources)<br>Anti-α-tubulin, mouse antibody (Sigma, T9026)<br>Anti-GFP, mouse antibody (Invitrogen, A-11122)<br>Anti-GFP, chicken antibody (Abcam, ab13970)<br>Anti-mouse IgG Alexa Fluor 568, goat antibody (Invitrogen, A-11004)<br>Anti-rabbit IgG Alexa Fluor 488, goat antibody (Invitrogen, A-11008)<br>Anti-mouse IgG Alexa Fluor 594, goat antibody (Invitrogen, A-11014)<br>Anti-mouse IgG Alexa Fluor 488, goat antibody (Invitrogen, A-11001)<br>Anti-rabbit IgG Alexa Fluor 594, goat antibody (Invitrogen, A-11012)<br>Anti-mouse IgG Alexa Fluor 488, goat antibody (Invitrogen, A-11034)<br>Anti-rabbit IgG Alexa Fluor 568, goat antibody (Invitrogen, A-11011)<br>Anti-chicken IgG Alexa Fluor 488, goat antiody (invitrogen, A-11039)
Validation	All commercial antibodies used in this study have been validated by the supplier.<br>Anti-PfGAP, rabbit antibody were validated by Thomas et al, 2012; PLOS One.

Eukaryotic cell lines

Policy information about cell lines and Sex and Gender in Research

Cell line source(s)	The work performed in this work was on strains of Plasmodium falciparum which were obtained from MR4 and BEI and Plasmodium berghei ANKA line 2.34 and ANKA line 507cl1 expressing GFP, which were subsequently genetically modified and the validation is reported in the manuscript.
Authentication	BEI and MR4 for P. falciparum strains 1G5DC. Billker et al, 2004; Cell for P. berghei ANKA line 2.34 and Janse et al, 2006; Molecular and Biochemical Parasitology for P. berghei ANKA line 507cl1 expressing GFP. Details provided in the Methods/ Suppl Information.
Mycoplasma contamination	NA
Commonly misidentified lines<br>(See ICLAC register)	NA

Animals and other research organisms

Policy information about studies involving animals; ARRIVE guidelines recommended for reporting animal research, and Sex and Gender in Research

Laboratory animals	Six- to eight-week-old female CD1 outbred mice from Charles River laboratories were used for all experiments. The conditions of mice kept are a 12hour light and 12hour dark (7 till 7) light cycle, the room temperature is kept between 20-24 degrees Celcius and the humidity is kept between 40-60%.
Wild animals	NA
Reporting on sex	In this study it was not really important to choose female or male mice because we used them as a media/vehicle to grow Plasmodium and did not study the effect of infection on mice. The important part was to grow the parasite in mice with sustainable

parasitaemia that could be easily handled and managed. The Plasmodium grow exactly same way in both sexes, that we have tested previously. We used here female mice without any specific reason but found them less aggressive and easy to handle.

Field-collected samples: No field collected samples were used in the study.

Ethics oversight: The animal work passed an ethical review process and was approved by the United Kingdom Home Office. Work was carried out under UK Home Office Project Licenses (PDD2D5182 and PP3589958) in accordance with the UK 'Animals (Scientific Procedures) Act 1986'.

Note that full information on the approval of the study protocol must also be provided in the manuscript.

Plants

Seed stocks: NA

Novel plant genotypes: NA

Authentication: NA

Flow Cytometry

Plots

Confirm that:

- [ ] The axis labels state the marker and fluorochrome used (e.g. CD4-FITC).
- [ ] The axis scales are clearly visible. Include numbers along axes only for bottom left plot of group (a 'group' is an analysis of identical markers).
- [ ] All plots are contour plots with outliers or pseudocolor plots.
- [x] A numerical value for number of cells or percentage (with statistics) is provided.

Methodology

Sample preparation: Described in the methods section
For determining parasitemia using flow cytometry, samples were fixed with 1%PFA and 0.0075% glutaraldehyde solution and kept on an end-to-end rocker for 15min. After completion, samples were either stored at 4oC or processed directly for Hoechst 33342 staining for 10-min at 37ºC.

Instrument: BDverse (BD Biosciences) or BD FACSymphony A1 Flow Cytometers

Software: FlowJo v10

Cell population abundance: We used flow cytometry to detect the Plasmodium falciparum parasitemia levels in human red blood cells, we did not use the flow cytometry to sort the specific cells

Gating strategy: as indicated in relevant reference Theron et al 2010 and Rawat et al 2025

- [ ] Tick this box to confirm that a figure exemplifying the gating strategy is provided in the Supplementary Information.
